# Distributed Field Estimation Using Sensor Networks Based on *H*_∞_ Consensus Filtering

**DOI:** 10.3390/s18103557

**Published:** 2018-10-20

**Authors:** Haiyang Yu, Rubo Zhang, Junwei Wu, Xiuwen Li

**Affiliations:** 1 Key Laboratory of Intelligent Perception and Advanced Control of State Ethnic Affairs Commission, Dalian Minzu University, Dalian 116600, China; yuhy@dlnu.edu.cn (H.Y.); wujunwei@dlnu.edu.cn (J.W.); 2 College of Science, Dalian Minzu University, Dalian 116600, China

**Keywords:** field estimation, *H*_∞_ filtering, consensus filtering, finite element method

## Abstract

This paper is concerned with the distributed field estimation problem using a sensor network, and the main purpose is to design a local filter for each sensor node to estimate a spatially-distributed physical process using the measurements of the whole network. The finite element method is employed to discretize the infinite dimensional process, which is described by a partial differential equation, and an approximate finite dimensional linear system is established. Due to the sparsity on the spatial distribution of the source function, the $\ell_{1}$-regularized $H_{\infty }$ filtering is introduced to solve the estimation problem, which attempts to provide better performance than the classical centralized Kalman filtering. Finally, a numerical example is provided to demonstrate the effectiveness and applicability of the proposed method.

## 1. Introduction

Many spatially-distributed physical phenomena are modeled as scalar or vector fields, which are governed by partial differential equations (PDEs), e.g., the distribution of temperature, the concentration of pollutants in atmosphere or water and the dynamics of flows. Monitoring these physical processes is an important issue in various engineering areas, e.g., fire protection, localization of sound or pollutant sources. The recent advances in wireless sensor network technology provide a useful tool to perform these tasks. Due to the constraints on energy and communication bandwidth of a single sensor node, distributed information processing is usually employed in wireless sensor networks. To obtain a valid estimate of the spatially-distributed field, the main challenges are designing effective distributed algorithms to fuse the measurement data of the whole network.

During the past decade, the distributed estimation or filtering problems with sensor networks had been paid much attention, and there are many researchers who have worked on these issues [1,2]. Consensus filtering is the most widely-discussed data fusion method. In [3], three types of distributed Kalman filtering algorithms have been proposed. A distributed high-pass consensus filter was used to fuse local measurements, such that all sensor nodes could track the average measurement of the whole network. These algorithms are established based on the information form of Kalman filtering, and analyses of the stability and performance of the Kalman-consensus filter have been provided in [4]. The optimal Kalman filter was extended to three improved distributed algorithms in [5] by employing data-driven transmission schemes to reduce communication expenses. In [6], the distributed state estimation problem of nonlinear systems was discussed by means of the consensus extended Kalman filtering, and the stability analysis of the proposed algorithms was provided. A distributed Kalman consensus filter was developed in [7] for continuous-time dynamic systems based on a novel information weight method, so that the estimates on all sensor nodes converged to consensus values. The distributed estimation problems were discussed for mobile sensor networks in [8], and a two-stage extended Kalman-consensus filter algorithm was proposed.

In recent years, distributed filtering algorithms have been developed to solve field estimation problems. A reduced-order Kalman–Bucy filter was constructed in [9] to estimate a time-varying random field, so that the communication costs and root mean squared error could reach the Pareto optimality. In [10], the acoustic source tracking problem was considered, and a distributed algorithm was established based on particle filter. A distributed sparse Bayesian learning algorithm was developed in [11] based on variational inference and loopy belief propagation to estimate a spatial field.

Since the spatially-distributed physical process is modeled as an infinite dimensional system, spatial discretization techniques are usually employed to reduce it to a finite dimensional linear system, and the finite element method is the most commonly-used technology. The static field estimation problem with an unknown source function was discussed in [12], and a constrained optimization problem with a $\ell_{1}$-regularization term was built by means of the sparsity on the spatial distribution of the point sources. A distributed implementation of the proposed algorithm was developed in [13]. In [14], the spatial domain was decomposed into some overlapping subdomains to assign a communication network, and the parallel Schwartz method was employed to form a consensus strategy for the local Kalman filters on each sensor node. A field estimation method was proposed in [15] based on the variational inverse and finite element method, and a distributed query system was designed to provide an estimate anywhere in the domain without requiring the full environment representation.

In this paper, we aim to solve the field estimation problems by combining distributed $H_{\infty }$ consensus filtering with the finite element method. By means of the sparsity on the spatial distribution of the point sources, a $\ell_{1}$-regularization term is integrated into the design of the $H_{\infty }$ filtering algorithm by introducing a pseudo-measurement equation to improve the performance. The remainder of this paper is organized as follows. Section 2 provides the setup of the spatially-distributed physical process, and a Dirichlet boundary value problem is built based on a Poisson equation. The Galerkin finite element method is employed to discretize the PDE, and an approximate finite dimensional linear system is established. In Section 3, the centralized $\ell_{1}$-regularized $H_{\infty }$ filtering is introduced, and a distributed implementation is developed based on an average consensus filter. A numerical example is given in Section 4 to demonstrate the effectiveness of the proposed method, and Section 5 presents some concluding remarks.

Notations: R denotes the real number set, and $R^{n}$ is the *n*-dimensional Euclidean space. Given $a,b\in R^{n}$, $a\cdot b=a^{T}b$ denotes the inner production. ${∥a∥}_{2}=\sqrt{x\cdot x}$ is the Euclidean norm. $\nabla =[\frac{\partial }{\partial x},\frac{\partial }{\partial y}]$ is the differential operator on $R^{2}$. $\Delta =\nabla^{2}$ is the Laplacian operator. $H_{0}^{1}\left(\Omega \right)$ is the Sobolev space on Ω.

## 2. Problem Formulation

Consider a Poisson equation in two-dimensional space:

(1) −Δu(x,y)=f(x,y),

where $\left(x,y\right)\in \Omega \subset R^{2}$, and Ω is a specific domain in the space. $u:R^{2}\to R$ is the scalar field function, and $f:R^{2}\to R$ is the source function. Specially, in this paper, we assume the field is generated by some point sources sparsely distributed in Ω, so

$$f\left(x,y\right)=\sum_{s}\delta \left(x-x_{s},y-y_{s}\right),$$

where δ(x,y) is the Kronecker function and $\left(x_{s},y_{s}\right)$ is the location of the point source, which is unknown. The Dirichlet boundary value problem is considered on (1), and assume zero boundary conditions without loss of generality to simplify the notation,

(2) u(x,y)=0for(x,y)∈∂Ω.

There are many physical phenomena that can be modeled as Poisson equations, e.g., stable temperature fields or static electric fields.

The measurements are provided by a set of sensors $\left\{S_{1},\cdots ,S_{m}\right\}$, which are deployed randomly in Ω, and the locations of sensors are assumed to be known, denoted as $(x_{i},y_{i}),\;i=1,\cdots ,m$. Each sensor can measure the field value for its own location successively and provides a sample series,

(3) $$z_{i,k}=u\left(x_{i},y_{i}\right)+w_{i,k},$$

where $\left\{w_{i,k}\right\}$ is a zero-mean white Gaussian sequence with variance $R_{i}>0$. The sensor node can communicate and exchange information with its adjacent nodes. The communication topology can be described as an undirected graph, and the adjacent node set of $S_{i}$ is denoted as $N_{i}$.

The main problem discussed in this paper is to estimate the field function u(x,y) using measurements $\{z_{i,k}\},\;i=1,\cdots ,m$. However, u(x,y) is an infinite dimensional function on Ω governed by the PDE (1), and there are no effective algorithm frameworks to deal with this spatially-distributed process directly. To solve this estimation problem, a mainstream treatment is discretizing the domain Ω and approximating the PDE with a finite dimensional linear system.

Finite element methods are the most popular discrete numerical technologies in various engineering problems. Here, we employ the famous Galerkin finite element method [16] to discretize Equation (1).

Firstly, transform (1) into the following weak formulation. Given

(4) $$\int_{\Omega }\left(\Delta u(x,y)+f(x,y)\right)v\left(x,y\right)d\Omega =0$$

for any $v\left(x,y\right)\in H_{0}^{1}\left(\Omega \right)$, where v(x,y) is called the test function. By means of integration by parts and the boundary condition (2), (4) can be rewritten as

(5) $$\int_{\Omega }\nabla u\left(x,y\right)\cdot \nabla v\left(x,y\right)d\Omega =\int_{\Omega }f\left(x,y\right)v\left(x,y\right)d\Omega .$$

Compare with (1), the weak formulation (5) relaxes the constraint on the differentiability of the solution u(x,y).

Construct a triangularization mesh M={P,T} to discretize the domain Ω, where $P=\{P_{1},\cdots ,P_{n}\}$ is the set of nodes, $T=\{T_{1},\cdots ,T_{s}\}$ is the set of triangles and $T_{\tau }\in T$ is a triangle formed by three nearest nodes in P. Assume $T_{\tau }\in T$ is formed by nodes $\left\{P_{\tau ,1},P_{\tau ,2},P_{\tau ,3}\right\}$. Define the linear basis functions on $T_{\tau }$ as

$$\begin{array}{ccc} \phi_{\tau ,1}\left(x,y\right) & = & a_{\tau ,1}x+b_{\tau ,1}y+c_{\tau ,1},\\ \phi_{\tau ,2}\left(x,y\right) & = & a_{\tau ,2}x+b_{\tau ,2}y+c_{\tau ,2},\\ \phi_{\tau ,3}\left(x,y\right) & = & a_{\tau ,3}x+b_{\tau ,3}y+c_{\tau ,3}, \end{array}$$

satisfying the following conditions

$$\begin{array}{c} \phi_{\tau ,1}\left(P_{\tau ,1}\right)=1,\;\phi_{\tau ,1}\left(P_{\tau ,2}\right)=0,\;\phi_{\tau ,1}\left(P_{\tau ,3}\right)=0,\\ \phi_{\tau ,2}\left(P_{\tau ,1}\right)=0,\;\phi_{\tau ,2}\left(P_{\tau ,2}\right)=1,\;\phi_{\tau ,2}\left(P_{\tau ,3}\right)=0,\\ \phi_{\tau ,3}\left(P_{\tau ,1}\right)=0,\;\phi_{\tau ,3}\left(P_{\tau ,2}\right)=0,\;\phi_{\tau ,3}\left(P_{\tau ,3}\right)=1, \end{array}$$

and for $\left(x,y\right)\in \Omega \backslash T_{\tau }$,

$$\phi_{\tau ,1}\left(x,y\right)=\phi_{\tau ,2}\left(x,y\right)=\phi_{\tau ,3}\left(x,y\right)=0.$$

As the locations of $\left\{P_{\tau ,1},P_{\tau ,2},P_{\tau ,3}\right\}$ are known, the coefficients in basis functions can be obtained by solving the linear systems.

Now, construct approximations of u(x,y) and f(x,y) on $T_{\tau }$ using the linear basis functions

(6) $$\hat{u}\left(x,y\right)=\sum_{i=1}^{3}u_{\tau ,i}\phi_{\tau ,i}\left(x,y\right),$$

(7) $$\hat{f}\left(x,y\right)=\sum_{i=1}^{3}f_{\tau ,i}\phi_{\tau ,i}\left(x,y\right),$$

where $u_{\tau ,i}$ and $f_{\tau ,i}$ are the coordinates of *u* and *f* on these basis functions.

Recall the weak formulation (5), for the arbitrariness of v(x,y), we set $v\left(x,y\right)=\phi_{\tau ,j}\left(x,y\right)$, j=1,2,3. Substitute u(x,y) and f(x,y) by (6) and (7) respectively,

(8) $$\sum_{i=1}^{3}u_{\tau ,i}\int_{\Omega }\nabla \phi_{\tau ,i}\cdot \nabla \phi_{\tau ,j}d\Omega =\sum_{i=1}^{3}f_{\tau ,i}\int_{\Omega }\phi_{\tau ,i}\phi_{\tau ,j}d\Omega$$

for j=1,2,3.

Denote $a_{ij}^{\tau }=\int_{\Omega }\nabla \phi_{\tau ,i}\cdot \nabla \phi_{\tau ,j}d\Omega$, $b_{ij}^{\tau }=\int_{\Omega }\phi_{\tau ,i}\phi_{\tau ,j}d\Omega$, $A_{\tau }={\left[a_{ij}^{\tau }\right]}_{3\times 3}$, $B_{\tau }={\left[b_{ij}^{\tau }\right]}_{3\times 3}$, $u_{\tau }={\left[u_{\tau ,1},u_{\tau ,2},u_{\tau ,3}\right]}^{T}$, $f_{\tau }={\left[f_{\tau ,1},f_{\tau ,2},f_{\tau ,3}\right]}^{T}$, we obtain the following linear system from (8),

(9) $$A_{\tau }u_{\tau }=B_{\tau }f_{\tau }.$$

Similarly, the linear system like (9) can be obtained for each T∈T. For the same node *P* in adjacent triangles, combine the corresponding matrix elements in $A_{\tau }$ and $B_{\tau }$, then the global linear system can be rewritten as follows

(10) Au=Bf,

where $A\in R^{n\times n}$ is called the stiffness matrix and $B\in R^{n\times n}$ is called the mass matrix. In order to meet the boundary condition (2), the corresponding elements of u should be set to zeros for P∈∂Ω. *A* and *B* are positive-definite matrices [16].

Now, the infinite system (1) can be approximated by the finite dimensional linear system (10), where $u\in R^{n}$ is the approximate field function and $f\in R^{n}$ is the approximate source function. Obviously, the field function u can be reconstructed by solving (10) if the source function f can be estimated effectively using the sensor measurements.

## 3. $H_{\infty }$ Consensus Filtering

Now, we attempt to estimate the approximate source function f by means of establishing a filtering problem.

Without loss of generality, assume the locations of all sensors are included in P. If $S_{i}$ is located at $P_{j}$, the measurement model (2) can be rewritten as following

(11) $$z_{i,k}=u_{j}+w_{i,k}=C_{i}f+w_{i,k},$$

where $C_{i}=\Delta_{i}A^{-1}B$, $\Delta_{i}={\left[\delta_{ij}\right]}_{1\times n}$, and

$$\begin{array}{c} \delta_{ij}=\left\{\begin{array}{cc} 1 & S_{i}\;\mathrm{locates}\;\mathrm{at}\;P_{j},\\ 0 & \mathrm{Otherwise}. \end{array}\right. \end{array}$$

Denote $C={\left[C_{1}^{T},\cdots ,C_{m}^{T}\right]}^{T}$, $z_{k}={\left[z_{1,k},\cdots ,z_{m,k}\right]}^{T}$, $w_{k}={\left[w_{1,k},\cdots ,w_{m,k}\right]}^{T}$, $R=\mathrm{diag}\{R_{1},\cdots ,R_{m}\}$, then the global measurement model can be defined as

(12) $$z_{k}=Cf+w_{k}.$$

Now, the field estimation problem can be converted to a filtering problem: estimate the unknown signal f using measurement series $\left\{z_{k}\right\}$. In general, the dimension of $z_{k}$ is much less than that of f. As mentioned in the previous section, the source term f(x,y) in (1) is assumed to be some point sources sparsely distributed in Ω, which means f is a sparse signal in $R^{n}$. Therefore, the $\ell_{1}$-regularized $H_{\infty }$ filtering algorithm proposed in [17] can be employed to solve the sparse signal estimation problem.

Construct a pseudo-measurement equation

0=Hf+ϵ,

where $H=\mathrm{sign}{\left(f\right)}^{T}$ is the pseudo-measurement matrix, $\epsilon ∼N(0,\sigma^{2})$, σ is a parameter referring to the sparsity constraint. According to Theorem 1 in [17], the source signal f can be estimated using the centralized $\ell_{1}$-regularized $H_{\infty }$ filtering algorithm by performing the following two procedures alternately based on global measurement (12).

Measurement update:

$$\begin{array}{ccc} \xi_{k} & = & C^{T}R^{-1}z_{k},\\ S^{\left[1\right]} & = & C^{T}R^{-1}C,\\ P_{k} & \leftarrow & {\left(P_{k}^{-1}+S^{\left[1\right]}\right)}^{-1},\\ {\hat{f}}_{k} & \leftarrow & {\hat{f}}_{k}+P_{k}\left(\xi_{k}-S^{\left[1\right]}{\hat{f}}_{k}\right), \end{array}$$

Pseudo-measurement update:

$$\begin{array}{ccc} S_{k}^{\left[2\right]} & = & \sigma^{-2}H_{k}^{T}H_{k},\\ P_{k} & \leftarrow & {\left(P_{k}^{-1}+S_{k}^{\left[2\right]}-\gamma^{-2}I\right)}^{-1},\\ {\hat{f}}_{k} & \leftarrow & \left(I-{\left(P_{k}^{-1}+\gamma^{-2}I\right)}^{-1}S_{k}^{\left[2\right]}\right){\hat{f}}_{k},\\ {\hat{u}}_{k} & = & A^{-1}B{\hat{f}}_{k}, \end{array}$$

where *I* is an identity matrix with proper dimensions, ${\hat{f}}_{k}$ is the estimate value of f at step *k*, ${\hat{u}}_{k}$ is the estimate value of u at step *k*, and γ is a parameter referring to the $H_{\infty }$ disturbance attenuation level. The pseudo-measurement matrix *H* is approximated by $H_{k}=\mathrm{sign}{\left({\hat{f}}_{k}\right)}^{T}$ since it is dependent on f, which is unknown.

To develop a distributed algorithm, establish the following local filter on each sensor node, which has the same performance as the centralized $\ell_{1}$-regularized $H_{\infty }$ filter [17].

Measurement update:

(13) $$\begin{array}{ccc} {\bar{\xi }}_{k} & = & \frac{1}{m}\sum_{i=1}^{m}C_{i}^{T}R_{i}^{-1}z_{i,k}, \end{array}$$

(14) $$\begin{array}{ccc} {\bar{S}}^{\left[1\right]} & = & \frac{1}{m}\sum_{i=1}^{m}C_{i}^{T}R_{i}^{-1}C_{i}, \end{array}$$

(15) $$\begin{array}{ccc} P_{i,k} & \leftarrow & {\left(P_{i,k}^{-1}+{\bar{S}}^{\left[1\right]}\right)}^{-1}, \end{array}$$

(16) $$\begin{array}{ccc} {\hat{f}}_{i,k} & \leftarrow & {\hat{f}}_{i,k}+P_{i,k}\left({\bar{\xi }}_{k}-{\bar{S}}^{\left[1\right]}{\hat{f}}_{i,k}\right), \end{array}$$

Pseudo-measurement update:

(17) $$\begin{array}{ccc} S_{i,k}^{\left[2\right]} & = & \sigma^{-2}H_{i,k}^{T}H_{i,k}, \end{array}$$

(18) $$\begin{array}{ccc} P_{i,k} & \leftarrow & {\left(P_{i,k}^{-1}+S_{i,k}^{\left[2\right]}-\gamma^{-2}I\right)}^{-1}, \end{array}$$

(19) $$\begin{array}{ccc} {\hat{f}}_{i,k} & \leftarrow & \left(I-{\left(P_{i,k}^{-1}+\gamma^{-2}I\right)}^{-1}S_{i,k}^{\left[2\right]}\right){\hat{f}}_{i,k}, \end{array}$$

(20) $$\begin{array}{ccc} {\hat{u}}_{i,k} & = & A^{-1}B{\hat{f}}_{i,k}, \end{array}$$

where $H_{i,k}=\mathrm{sign}{\left({\hat{f}}_{i,k}\right)}^{T}$. Notice that (13) and (14) define the average measurement and the average inverse covariance matrix of the sensor network, which cannot be obtained directly by sensor nodes. By means of the following average consensus algorithm proposed in [4], all the local filters can approximate (13) and (14) gradually by communicating with adjacent nodes,

$$\begin{array}{ccc} \dot{q_{i}} & = & \beta \sum_{j\in N_{i}}\left(\left(q_{j}-q_{i}\right)+\left(u_{j}-u_{i}\right)\right),\\ \eta_{i} & = & q_{i}+u_{i}. \end{array}$$

It has been verified that, if the network topology is strongly connected,

$$\eta_{i}\to \frac{1}{m}\sum_{i=1}^{m}u_{i}\;\;\mathrm{as}\;\;t\to \infty ,\;\;\forall i\in \left\{1,\cdots ,m\right\}.$$

The distributed estimation algorithm is summarized as the following Algorithm 1.

**Algorithm 1** Distributed $H_{\infty }$ consensus filtering for field estimation.
1:**Initialization**: $q_{i,0}=0$, $Q_{i,0}=0$, $P_{i,0}=P_{0}$, ${\hat{f}}_{i,0}=f_{0}$.2:
**for** k=0,⋯,N **do**
3: Compute $u_{i,k}=C_{i}^{T}R_{i}^{-1}z_{i,k}$, $U_{i,k}=C_{i}^{T}R_{i}^{-1}C_{i}$.4: Send $\left\{u_{i,k},q_{i,k},U_{i,k},Q_{i,k}\right\}$ to node $j\in N_{i}$.5: Receive $\left\{u_{j,k},q_{j,k},U_{j,k},Q_{j,k}\right\}$ from node $j\in N_{i}$.6: Fuse data using average consensus filter:
$$\begin{array}{ccc} q_{i,k+1} & = & q_{i,k}+\beta \sum_{j\in N_{i}}\left(\left(q_{j,k}-q_{i,k}\right)+\left(u_{j,k}-u_{i,k}\right)\right),\\ Q_{i,k+1} & = & Q_{i,k}+\beta \sum_{j\in N_{i}}\left(\left(Q_{j,k}-Q_{i,k}\right)+\left(U_{j,k}-U_{i,k}\right)\right),\\ {\hat{\xi }}_{i,k} & = & q_{i,k+1}+u_{i,k},\\ {\hat{S}}_{i,k}^{\left[1\right]} & = & Q_{i,k+1}+U_{i,k}. \end{array}$$7: Compute local measurement update using (15) and (16).8: Compute pseudo-measurement update using (18)–(20).9:
**end for**



## 4. Numerical Example

**Figure 1:**
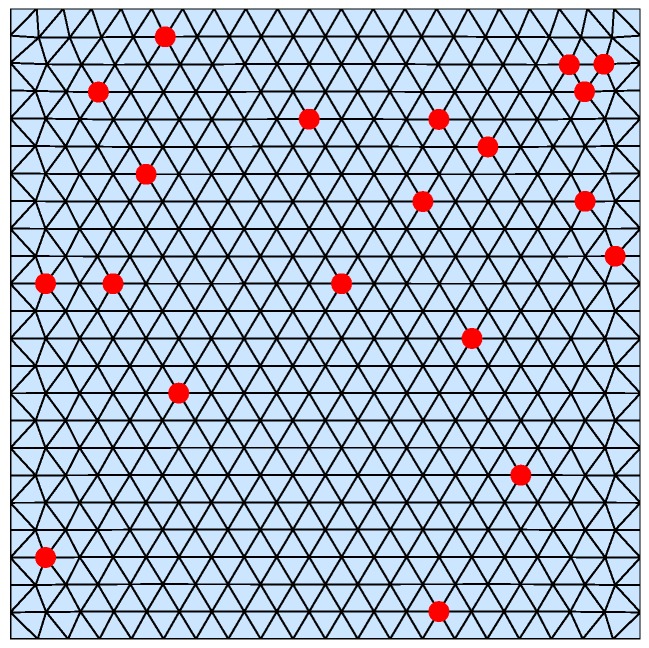
Mesh on Ω. The red points show the locations of sensors.

Consider a scalar field governed by a Poisson equation on a square domain Ω=[−1,1]×[−1,1]. A mesh M={P,T} is generated on Ω using Distmesh [18], which has 494 nodes and 899 triangles. That is, n=494, $P=\{P_{1},\cdots ,P_{494}\}$, $T=\{T_{1},\cdots ,T_{899}\}$. Deploy 20 sensors $\left\{S_{1},\cdots ,S_{20}\right\}$ randomly in Ω∖∂Ω, and the location set is $\{P_{402},P_{72},P_{181},P_{205},P_{162},P_{489},P_{486},P_{60},P_{239},P_{130},P_{456},P_{159},$ $P_{76},$ $P_{129},P_{70},P_{118},P_{241},P_{226},P_{439},P_{66}\}$. All sensors form a network by means of a fully-connected communication topology. The mesh and sensor nodes are shown in Figure 1.

The source function f(x,y) is defined as a single point source located at (0.5,0.5). Each sensor provides a measurement based on (2), and the standard deviations of measurement noises are set to 0.01. The initial states of local filters are ${\hat{f}}_{i,0}=0$, $P_{i,0}=I_{494}$, i=1,⋯,20. The parameters of local filters are β=0.015, γ=10, σ=120 and N=100.

Now, we are ready to solve the field estimation problem using Algorithm 1. The centralized Kalman filtering using global measurement (12) is employed as a benchmark, and the following normalized mean squared error (NMSE) is introduced to evaluate the performance,

$$e_{i}=\frac{∥u-{\hat{u}}_{i}{∥}_{2}}{{∥u∥}_{2}}.$$

Define the average consensus error (ACE)

$$\varepsilon_{i}=∥{\hat{u}}_{i}{∥}_{2}-\frac{1}{m}\sum_{j=1}^{m}{∥{\hat{u}}_{j}∥}_{2}$$

to evaluate the consensus level on the estimated field functions of all local filters.

**Figure 2:**
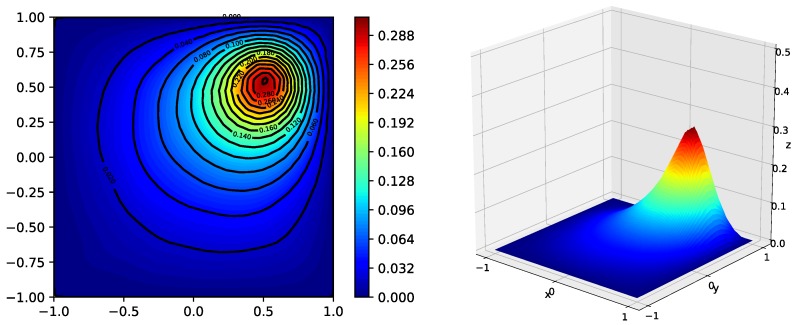
Estimated field function $\hat{u}$ outputs from the local filter on $S_{20}$ using Algorithm 1. The **left** figure shows $\hat{u}$ in the two-dimensional perspective with contour lines; the **right** figure shows $\hat{u}$ in the three-dimensional perspective.

**Figure 3:**
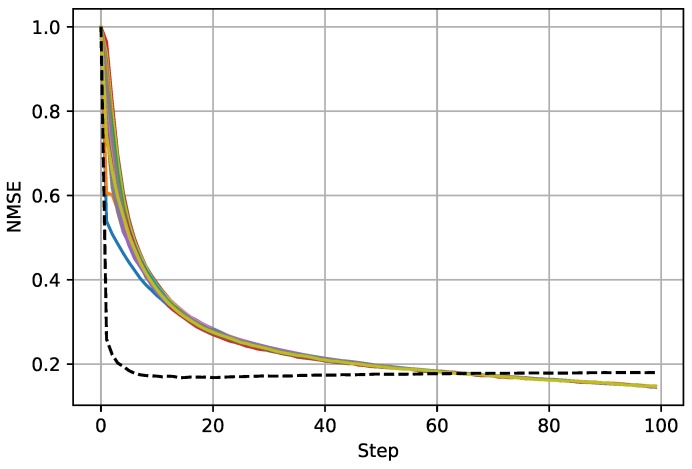
NMSEs of the centralized Kalman filter and local filters. The black dashed line presents the NMSE of centralized Kalman filter, and the solid lines present the NMSEs of 20 local filters.

**Figure 4:**
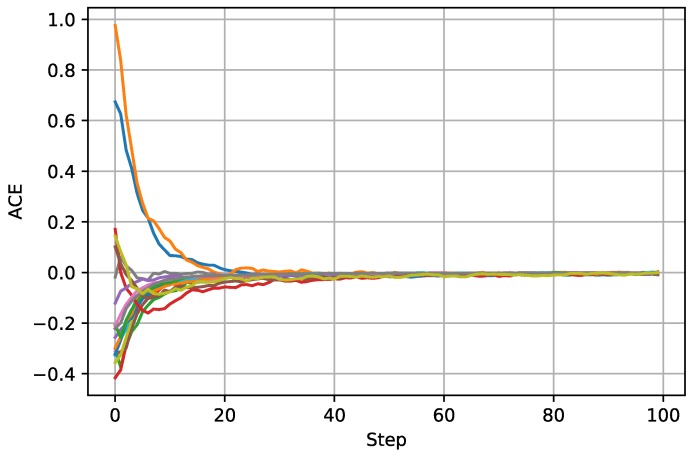
Average consensus errors (ACEs) of 20 local filters.

The simulation results are presented in Figure 2, Figure 3 and Figure 4. Figure 2 shows the estimated field function outputs from the local filter on $S_{20}$ with two pseudo-color images. The left figure demonstrates the scalar field in the two-dimensional perspective with contour lines. The right figure demonstrates the same result in the three-dimensional perspective, where the *z*-coordinate represents the value of the field function. Figure 3 presents the normalized mean squared errors, showing that all local filters using Algorithm 1 are stable. Moreover, the steady-state errors of all local filters are smaller than that of centralized Kalman filtering, which indicates that the sparseness constraint condition on the source function can improve the performance of local filters. The NMSEs of centralized Kalman filtering and local filters are presented in Table 1. As shown in Figure 4, the average consensus errors converge to zero, which means that all local filters provide the same estimated results as $S_{20}$. All these results demonstrate the effectiveness of the proposed algorithm.

## 5. Conclusions

This paper investigated the scalar field estimation problem with the measurements from a sensor network. An infinite dimensional physical process governed by a Poisson PDE was considered. Based on the Galerkin finite element method, the infinite dimensional physical process was approximated by a finite dimensional linear system, and a filtering problem was established. By means of the sparsity of the point sources, a so-called $\ell_{1}$-regularized $H_{\infty }$ filtering was employed to solve the distributed estimation problem. It was illustrated in simulation that the proposed distributed filtering algorithm is effective and has better performance than centralized Kalman filtering. However, since the $\ell_{1}$-regularized $H_{\infty }$ filtering was developed based on compressive sampling theory, the restricted isometry property of the global measurement matrix used in this paper has not been verified. Moreover, a proper decomposition of the stiffness and mass matrices can improve the computational performance of the proposed algorithm. The results of this work can be applied to the localization or tracking of sources. These problems will be discussed in our future works.

## Figures and Tables

**Table 1:** NMSEs of the centralized Kalman filter and local filters using Algorithm 1.

**Algorithm**	**Centralized KF**	**Node 1**	**Node 2**	**Node 3**	**Node 4**	**Node 5**	**Node 6**
NMSE	0.1771	0.1445	0.1470	0.1460	0.1458	0.1446	0.1457
**Algorithm**	**Node 7**	**Node 8**	**Node 9**	**Node 10**	**Node 11**	**Node 12**	**Node 13**
NMSE	0.1462	0.1459	0.1461	0.1547	0.1457	0.1461	0.1456
**Algorithm**	**Node 14**	**Node 15**	**Node 16**	**Node 17**	**Node 18**	**Node 19**	**Node 20**
NMSE	0.1461	0.1438	0.1449	0.1456	0.1453	0.1463	0.1449
